# The duration of balloon inflation affects the luminal diameter of coronary segments after bioresorbable vascular scaffolds deployment

**DOI:** 10.1186/s12872-015-0163-5

**Published:** 2015-12-11

**Authors:** Sabato Sorrentino, Salvatore De Rosa, Giuseppe Ambrosio, Annalisa Mongiardo, Carmen Spaccarotella, Alberto Polimeni, Jolanda Sabatino, Daniele Torella, Gianluca Caiazzo, Ciro Indolfi

**Affiliations:** Division of Cardiology, Department of Medical and Surgical Sciences, Magna Graecia University, Catanzaro, Italy; URT-CNR, Magna Graecia University, Catanzaro, Italy; Department of Medical and Surgical Sciences and Director, URT Consiglio Nazionale delle Ricerche (CNR); Magna Graecia University, Catanzaro, 88100 Italy

**Keywords:** Bioresorbable vascular scaffolds, Deployment, Implantation technique, QCA, Coronary artery disease

## Abstract

**Background:**

Adequate expansion is critical to achieve optimal Bioresorbable Vascular Scaffolds (BVS) apposition to the vessel wall. However, compared to metallic stents, BVS present different mechanical properties. Hence, slow deployment and maintenance of balloon inflation for at least 30” is recommended for BVS implantation. However, since no evidences are available demonstrating the superiority of a longer balloon dilatation time, the implantation technique is highly variable among different centers.

**Methods:**

A total of 24 BVS-treated lesions were included in the present analysis. After BVS deployment at 12 atmosphere (ATM) the balloon was rapidly deflated and scaffold expansion was documented with an angiogram. The same balloon was then inflated again and kept at 12 ATM for 30”. Finally, a further angiogram was obtained to evaluate BVS expansion. Quantitative coronary angiography (QCA) was performed at each step.

**Results:**

A significant increase of minimal luminal diameter (MLD)-to-reference scaffold diameter (RSD) ratio (MLD to RSD Ration, MR-Ratio) from 0.70 ± 0.10 after initial stent deployment to 0.79 ± 0.10 after the 30”-long balloon dilation was observed (*p* < 0.001). Of note, this result was consistent across all sub-segments, as well as across almost all lesion subgroups. A substantial reduction in the prevalence of residual stenosis from 29 % to 17 % was registered after the 30”-long dilation.

**Conclusions:**

Our results strongly support the maintenance of balloon inflation for at least 30” during BVS deployment to achieve optimal scaffold expansion and minimize the occurrence of residual stenosis.

**Electronic supplementary material:**

The online version of this article (doi:10.1186/s12872-015-0163-5) contains supplementary material, which is available to authorized users.

## Background

Bioresorbable vascular scaffolds (BVS) are a promising new development with the potential to overcome several limitations of permanent metallic coronary stents [1–4]. In fact, the special chemical properties of the poly-L-lactic acid (PLLA) polymer allow complete struts resorption with multiple potential benefits, including restoration of normal vasoreactivity [4–6], preservation of physiologic vascular remodeling and reduction of the risk of late stent thrombosis [2, 3]. In addition, struts resorption preserves the possibility of a future surgical myocardial revascularization. Finally, the intrinsic properties of the scaffold allow undisturbed imaging with computer tomography and magnetic resonance.

It is well known that prolonged balloon inflation is superior to a rapid inflation/deflation during implantation of metallic stents [7]. Since bioresorbable scaffolds do not have the same radial strength observed with metallic stents, an optimal implantation technique may be even more important to achieve maximal expansion and prevent strut malapposition [8, 9]. Furthermore, given the almost doubled strut thickness of BVS compared to metallic scaffolds, it is important to achieve the largest possible luminal area to warrant the best fluido-dynamic conditions and prevent the risk for restenosis and stent thrombosis [10–12]. For these reasons, the producer suggests the following implantation protocol: slow BVS release with stepwise balloon inflation going up whit 2 ATM every 5 seconds, until the scaffold is completely expanded. At this point, the inflation pressure must be maintained for at least 30”. Finally, post-dilatation is possible at operator’s discretion whit either the delivery system, or with a non-compliant balloon (up to 0.5 mm larger than the implanted scaffold), to pursue optimal scaffold implantation. Accordingly, gradual scaffold deployment “pressurizing the delivery system in 2 ATM increments every five seconds” was also recommended in a recently published perspective document on technical aspects in PCI with BVS [13]. This recommendation was only supported by expert consensus, since no evidence is available, yet. This is an important issue, since the 30”-long balloon inflation step suggested by the producer is underused in few laboratories for several reasons, including the lack of published studies demonstrating the importance of such approach. If optimal stent implantation is undoubtedly effective in reducing the risk of malapposition for metallic platform, the implantation technique for bioresorbable scaffold is even more important, given the mechanical properties of BVSs. In light of the key importance of an optimal implantation technique, and in consideration of the still limited experience with such device, aim of the present studies was to evaluate the influence of a 30"-long balloon inflation on scaffold expansion, in coronary lesions treated with percutaneous angioplasty and implantation of an Absorb.

## Methods

### Bioresorbable vascular scaffold

The commercially available bioresorbable balloon expandable device (1.1 BVS revision, Abbott Vascular, Santa Clara, CA, USA) was used in the present study. It consists of a polymer backbone of poly-L-lactide (PLLA) coated with a thin layer of a 1:1 mixture of an amorphous matrix of poly-D,L-lactide (PDLLA), and 100 micrograms/cm_2_ of the anti-proliferative drug everolimus. The scaffold is radiolucent, but is provided with a platinum marker at each end that allows its visualization at fluoroscopy. Both PLLA and PDLLA are fully resorbable. The time for complete absorption of the polymer backbone is predicted to be about 2 years from preclinical studies however it can take longer times in humans [14]. The scaffold consists of 150 μm thick-struts arranged as in-phase zigzag hoops linked together by three longitudinal bridges. The device can be stored at room temperature.

### Study design and population

Consecutive coronary artery lesions treated with a second generation BVS (Abbott Vascular, Santa Clara, CA, USA) at the Division of Cardiology of the university Magna Graecia, Italy, in patients fulfilling the inclusion criteria, were included in the present analysis. Only lesions with adequate angiographic result after pre-dilation were selected for the study. The BVS was implanted through a stepwise balloon dilation, consisting in 2 ATM increments every 5 seconds up to 12 ATM. At this point the balloon was rapidly deflated and stent expansion documented with an angiogram. Then, the same balloon was quickly inflated again and kept at 12 ATM for 30 seconds. At this point the balloon was newly deflated and a further angiogram was obtained to re-evaluate BVS expansion. Any further post-dilatation was left at the operator’s discretion. The primary study endpoint was the comparison of minimal scaffold diameter to reference stent diameter ratio (MR-Ratio) before and after the 30“-long balloon dilation. Based on previous data from the available literature, we calculated that a sample size of 24 lesions would have provided a 90 % power whit a 5 % -error. Secondary endpoints were: the difference in luminal diameter in all scaffold sub-segments (proximal edge, proximal minimum diameter, central minimum diameter, distal minimum diameter and distal edge) before and after the 30”-long balloon dilation. The study protocol was approved by the local Ethics Review Board (Comitato Etico Regionale, Sezione Area Centro, Regione Calabria). A written consent was obtained by all patients.

### Quantitative coronary angiography

Scaffold expansion was measured at all time points using quantitative coronary angiography (QCA). For this aim, coronary angiograms were obtained after lesion predilatation, and after every step of scaffold deployment. For all angiograms, 8 ml (right coronary artery) or 12 ml (left coronary artery) of the contrast agent iomerol (Iomeron400®, Bracco Imaging) was injected with the use of a power injector (ACIST CMS2000, Bracco Imaging) at 3 ml/s (right coronary artery) or 4 ml/s (left coronary artery) injection rate with a pressure of 460 psi (right coronary artery) or 605 psi (left coronary artery). QCA was performed offline by two independent operators using angiograms that had passed the quality check (Additional file 1: Figure S1). In particular, a rapid and complete filling of the epicardial segment with the contrast agent was an important selection criteria for this study. Reference diameter (RD), minimal luminal diameter (MLD), minimal luminal diameter to reference scaffold diameter ratio (MR-Ratio), proximal edge diameter, distal edge diameter and percentage diameter stenosis were calculated offline. Optimum scaffold deployment according to QCA was defined as a residual diameter stenosis of less than 10 %.

### Statistical analysis

Continuous variables were expressed as a mean ± standard deviation, while categorical variables were presented as counts (%). The Wilcoxon test was used for comparison of continuous variables between paired groups, including the primary endpoint analysis. A *p* value of 0.05 was set as the threshold for statistical significance. All calculations were made with SPSS 15 software version (SPSS, Chicago IL) or Prism 5.0c.

## Results

### Baseline characteristics

A total of 24 consecutive lesions treated whit a BVS (Absorb) in 22 patients from July 2013 to January 2014 fulfilling the inclusion criteria were selected for the present study. Baseline patients’ characteristics and basal cardiovascular risk profile are reported in Table 1. Of all included patients, 9 (39 %) had stable angina (CAD), while 13 (61 %) presented an Acute Coronary Syndrome (ACS). In this last group 4 patients had unstable angina (UA), 7 had a non-ST-elevation Myocardial Infarction (NSTEMI) and 2 an ST-elevation Myocardial Infarction (STEMI). Angiographic population characteristics are shown in Table 2.

**Table 1:** Patient demographics characteristics (22 patients)

Age (mean ± SD; median)	61 ± 8.2; 60.5
Males (%)	17 (77 %)
Stable Angina (%)	9 (39 %)
Acute Coronary Sindrome	13 (61 %)
Unstable Angina (%)	4 (18 %)
NSTEMI (%)	7 (32 %)
STEMI (%)	2 (9 %)
Hypertension (%)	16 (72 %)
Hypercolesterolemia (%)	10 (45 %)
Diabetes Mellitus (%)	6 (27 %)
Smokers (%)	6 (27 %)
Previous AMI (%)	6 (27 %)
Previus-PTCA (%)	5 (23 %)
Cronic Kidney Disease (%)	2 (9 %)

*NSTEMI* non-ST segment elevation acute myocardial infarction, *STEMI* ST segment elevation acute myocardial infarction, *PTCA* percutaneous transcatheter coronary angioplasty

**Table 2:** Procedural characteristics (*n* = 24)

Target vessel		LAD	8
		LCX	11
		RCA	5
Disease extension	SVD	CAD	5
		ACS	12
	MVD	CAD	4
		ACS	2
RVD	≥2,5 mm		8
	<2,5 mm		16
Balloon Ratio	≥1		9
	<1		15
Balloon Pre-dilatation Length	≥12 mm		15
	<12 mm		9
Scaffold Diameter	2,5 mm		12
	3 mm		8
	3,5 mm		4
Scaffold Length	≥20 mm		4
	<20 mm		20

*LAD* left anterior descendant, *LCX* left circumflex artery, *RCA* right coronary artery, *SVD* single-vessel disease, *CAD* stable coronary artery disease, *ACS* acute coronary syndrome, *MVD* multiple-vessel disease, *RVD* reference vessel diameter

### Scaffold expansion

**Fig. 1:**
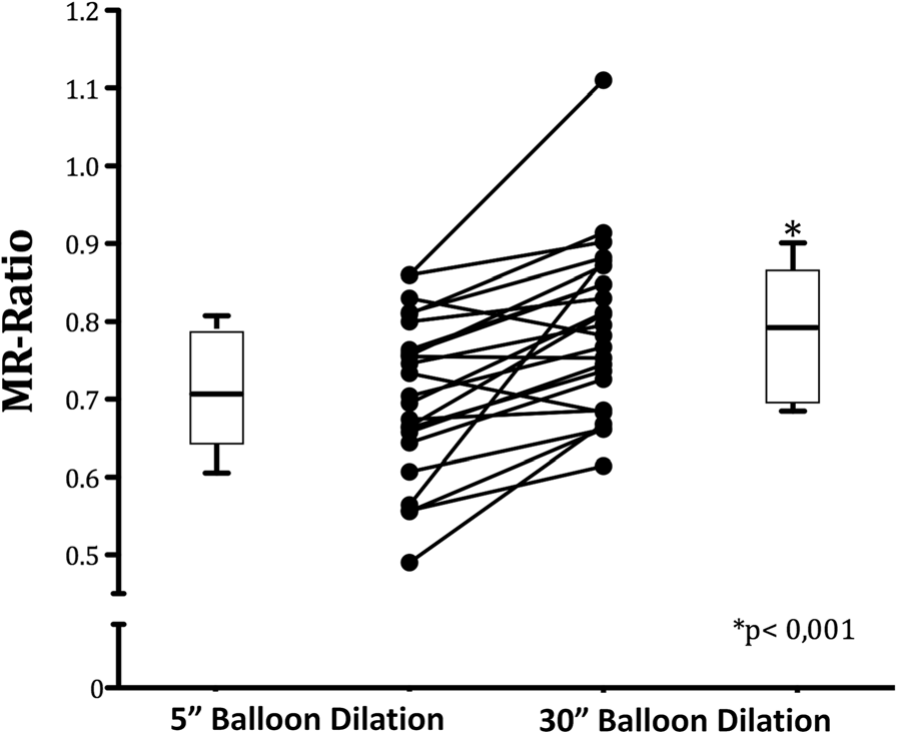
MLD-to-nominal scaffold diameter Ratio (MR-Ratio) before and after the 30”-long balloon dilation. Points represent pre- and post-dilation values for each lesion. Within the boxplots the mean (horizontal line) the interquartile range (box ends) and the 95 % Confidence Interval (whiskers) are reported

**Fig. 2:**
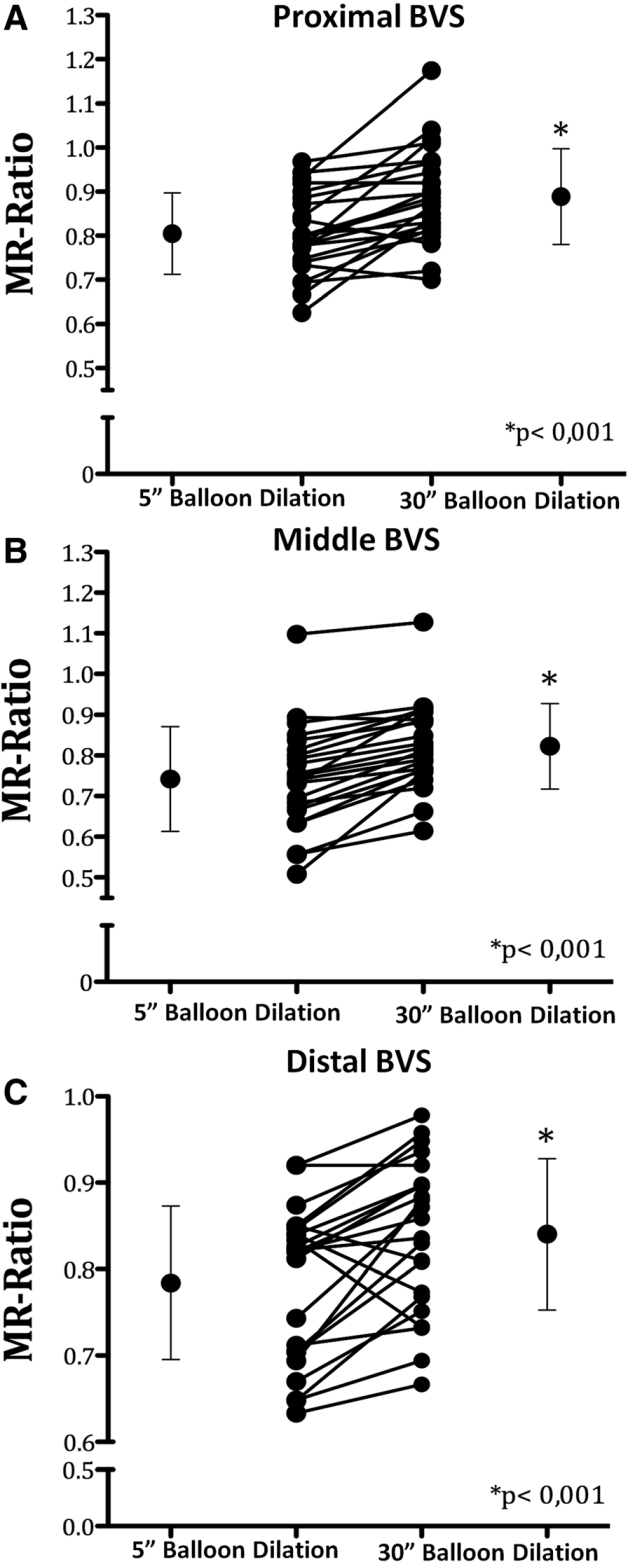
Mean MLD-to-nominal scaffold diameter Ratio (MR-Ratio) before and after the 30”-long balloon dilation for each scaffolded sub-segments: proximal (panel **a**) median (panel **b**) and distal (panel **c**)

**Fig. 3:**
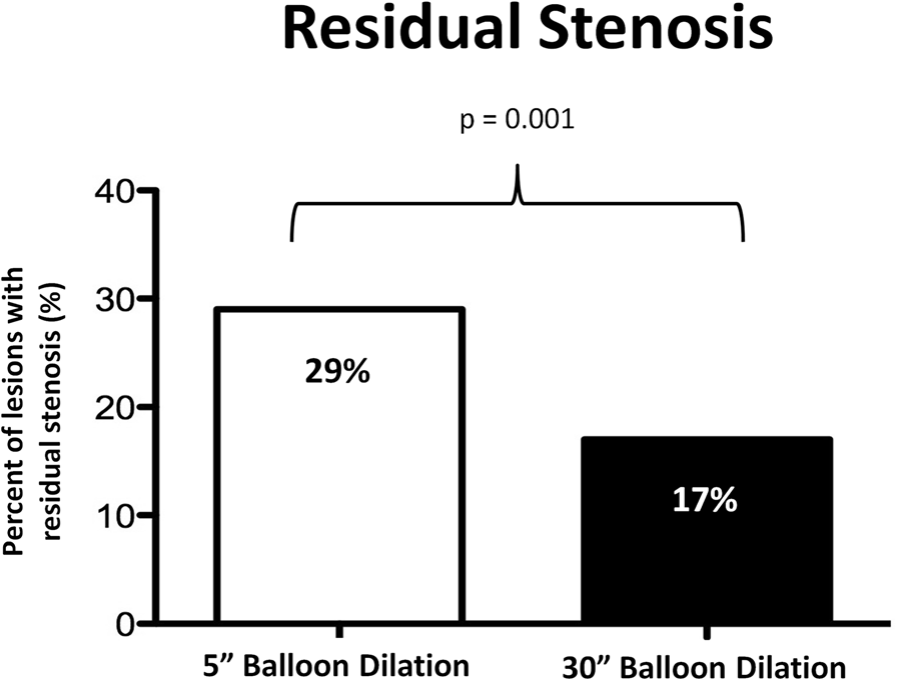
Prevalence of >10 % residual stenosis before (white bar) and after (black bar) the 30”-long balloon dilation

A significant increase of the minimal luminal diameter (MLD) to reference scaffold diameter (RSD) ratio (MR-Ratio) from 0.70 ± 0.10 after initial stent deployment to 0.79 ± 0.10 (*p* < 0.001) after the 30”-long balloon dilation was observed (Table 3; Fig. 1). Of note, this result was consistent across all sub-segments, even though this effect was more pronounced within the central segment (Table 3; Fig. 2). Most important, these results were associated with a substantial reduction in the prevalence of residual stenosis from 29 % after BVS deployment to 17 % after the 30 %-long dilation (*p* = 0.001) (Fig. 3).

**Table 3:** MR-Ratio scaffold measurement (*n* = 24)

QCA Variables	Deployment (MR-Ratio)	Long dilation (MR-Ratio)	Increase	*p* value
Minimal lumen diameter (mean; SD)	0.70 ± 0.10	0.79 ± 0.10	9 %	<0.001
Proximal MLD scaffold diameter (mean; SD)	0.80 ± 0.09	0.87 ± 0.11	7 %	<0.001
Central MLD scaffold diameter (mean; SD)	0.76 ± 0.12	0.83 ± 0.11	7 %	<0.001
Distal MLD scaffold diameter (mean; SD)	0.79 ± 0.08	0.84 ± 0.08	5 %	<0.001
Proximal edge (mean; SD)	0.88 ± 0.18	0.95 ± 0.16	7 %	<0.001
Distal edge (mean; SD)	0.80 ± 0.09	0.84 ± 0.11	4 %	<0.001

*MLD* minimal luminal diameter, *MR*-Ratio = *MLD*-to-reference scaffold diameter Ratio; *SD* standard deviation

**Fig. 4:**
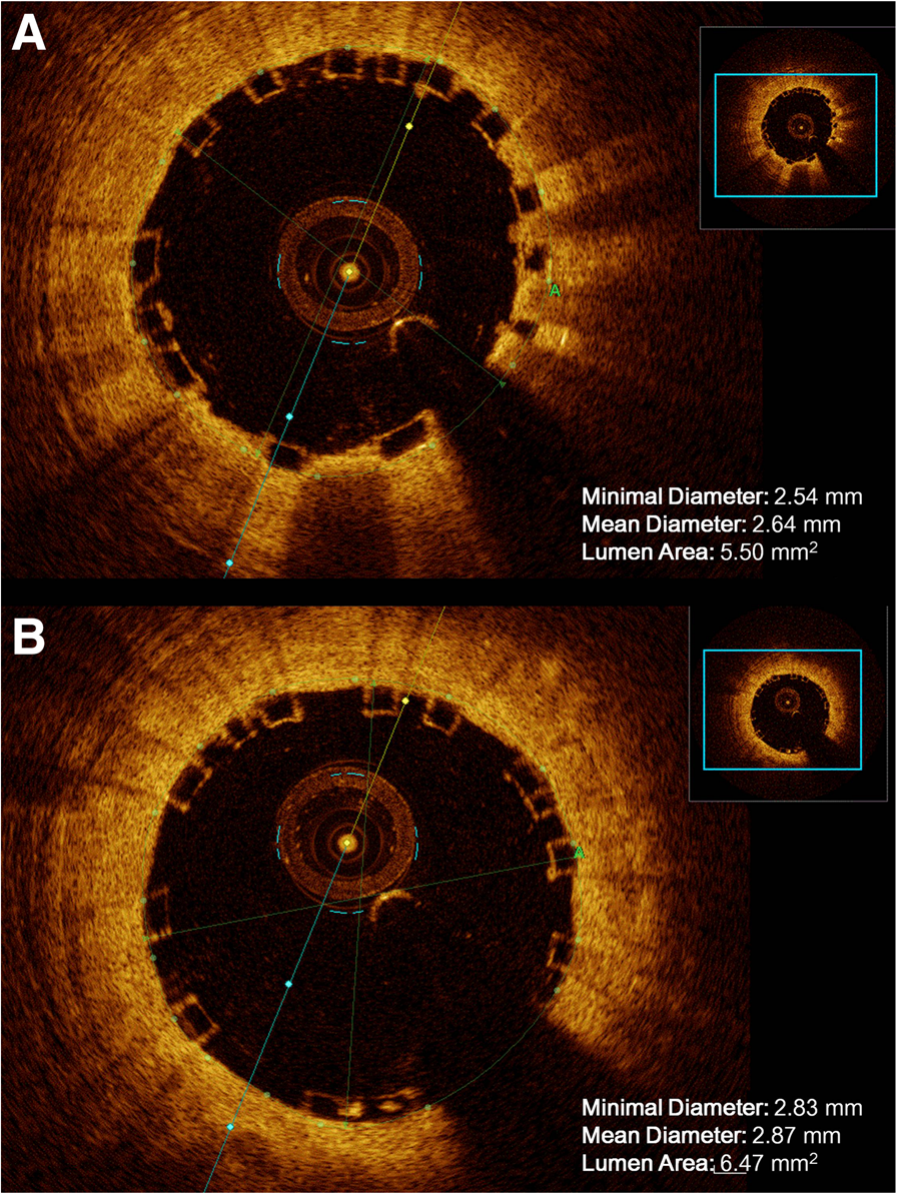
Illustrative example of the measurements obtained through OCT. Minimal Diameter, Mean Diameter and Lumen Area of Bioresorbable vascular scaffolds (BVS): before (a) and after (b) the 30”-long balloon dilation

Intracoronary imaging by means of optical coherence tomography (OCT) was obtained in a small series of three further patients, showing similar results as those obtained by means of QCA. In fact, MR-Ratio was increased from 0,83 ± 0,08 after BVS deployment to 0,93 ± 0,12 after the 30”-long balloon dilation. Interestingly, OCT analysis revealed a 33 % relative reduction in the number of malapposed struts after the 30”-long BVS dilation. Representative results with OCT imaging are reported in Fig. 4.

In the group with a nominal scaffold diameter (NSD) of 2.5 mm, the mean MLD increased from 2.03 ± 0.27 to 2.22 ± 0.32 within the proximal segment (*p* = 0.003), from 1.97 ± 0.4 to 2.13 ± 0.31 mm within the central segment (*p* = 0.003) and from 2.00 ± 0.21 to 2.14 ± 0.20 mm within the distal segment (*p* = 0.025). In the group with a NSD of 3.0 mm, the mean MLD increased from 2.49 ± 0.25 to 2.68 ± 0.34 mm within proximal segment (*p* = 0.014), from 2.3 ± 0.28 to 2.54 ± 0.36 mm within the central segment (*p* = 0.008) and from 2.48 ± 0.31 to 2.61 ± 0.27 mm within the distal segment (*p* = 0.014). Finally, in the group with a NSD of 3.5 mm, the mean MLD increased from 2.71 ± 0.17 to 2.95 ± 0.14 mm within the proximal segment (*p* = 0.125), from 2.46 ± 0.46 to 2.70 ± 0.57 mm the central segment (*p* < 0.125) and from 2.68 ± 0.42 to 2.82 ± 0.32 mm within the distal segment (*p* = 0.125) The lack of statistical significance within the group with a NSD of 3.5 mm is clearly due to the number of lesion in this group (Table 4; Additional file 2: Figure S2).

**Table 4:** MLD in all sub-segments (*n* = 24)

MLD (mean, SD)	Sub-segment	Deployment	Long dilation	*p* value
	Proximal	2.03 ± 0.27	2.22 ± 0.32	0.003
2.5 mm (n = 12)	Central	1.97 ± 0.4	2.13 ± 0.31	0.003
	Distal	2.0 ± 0.21	2.14 ± 0.2	0.025
	Proximal	2.49 ± 0.25	2.68 ± 0.34	0.014
3 mm (n = 8)	Central	2.3 ± 0.28	2.54 ± 0.36	0.008
	Distal	2.48 ± 0.31	2.61 ± 0.27	0.014
	Proximal	2.71 ± 0.17	2.95 ± 0.14	0.125
3.5 mm (n = 4)	Central	2.46 ± 0.46	2.70 ± 0.57	0.125
	Distal	2.68 ± 0.42	2.82 ± 0.32	0.125

*MLD* minimal luminal diameter, MR-Ratio = MLD-to-reference scaffold diameter Ratio, *SD* standard deviation

Furthermore, we also evaluated the effect of the 30”-long balloon dilation across different lesion subgroups (Additional file 2: Figure S2). Interestingly, the result of a better scaffold expansion after the 30”-long balloon dilatation was consistent across all lesion subgroups, with only two exception: lesion in which scaffold length was > 20 mm (*n* = 4) and lesions where a 3.5-diameter scaffold was implanted (*n* = 4). In these case the lack of a statistically significance may be related to the limited number of lesions in these subgroups. Finally, at multivariable analysis renal insufficiency (*p* = 0.021) and diabetes (*p* = 0.037) were the only independent predictors of the acute gain after the 30”-long dilation, while neither the balloon-to-BVS ratio (*p* = 0.726), nor hypercholesterolemia (*p* = 0.362), nor the presence of an ACS (*p* = 0.273) were significant predictors.

#### Scaffold post-dilation

Although the impact of post-dilation was not a direct objective of the present study, in 12 cases a post-dilation by means of a non-compliant balloon (NC balloon) was performed after scaffold implantation, at operator’s discretion. The diameter of the NC balloon was equal to the nominal scaffold diameter in 7 cases, and 0.25 mm larger than nominal scaffold diameter in 5 cases. Interestingly, the impact of additional post dilation by means of a non-compliant was very limited. In fact, the further increase of the MR-ratio was virtually comparable at the proximal (0,85 ± 0,11 to 0,885 ± 0,09; *p* = ns), the central (0,80 ± 0,08 to 0.81 ± 0,09; *p* = ns) and the distal (0,83 ± 0,07 to 0.85 ± 0,08; *p* = ns) segments.

## Discussion

The main finding of the present study is that duration of balloon inflation during the release of the device has a significant impact on the acute gain after BVS implantation. This result is particularly relevant, since the 30”-long dilation significantly improved scaffold expansion in all sub-segments and in almost all lesion subgroups. Our findings have practical relevance, as it is known that an optimal implantation technique is the key to prevent adverse events during the follow up.

In fact, among the major concerns with BVSs are scaffold under-expansion and acute elastic recoil of the vessel wall [9, 10, 14]. On the contrary, the present study revealed a good acute performance of BVS, when a proper implantation technique is applied to suitable lesions. This is an important confirmation for BVS efficacy, since both suboptimal deployment and a lower acute gain are associated to a higher incidence of target lesion failure and subsequent revascularization [15–18]. Of note the significant increase in the mean minimal lumen diameter documented in the present study after a longer balloon dilatation was responsible for a substantial reduction of the prevalence of residual stenosis from 29 % to 17 %. Even though our results only apply to a selected lesion subset, namely those that could be optimally pre-dilated, a proper implantation technique could be even more important in a broader lesion subset. However, specific studies are needed to evaluate the optimal implantation technique in a larger, unselected lesion set. Notwithstanding the initial enthusiasm around bioresorbable vascular scaffolds, that drove some centers to immediately reduce the selection criteria for BVS implantation, caution should be used, given the still limited experience with these devices. In particular, the bulky struts with larger luminal protrusion compared to metallic stents mandate maximal effort for prevention of malapposition. Of note, a lower renal function was associated to a lower increase in minimal scaffold diameter at multivariable analysis, in the present study. The concerns about an unrestricted use of BVS are also supported by the evidence that reabsorption time is actually longer than initially expected [19]. The present study fills a lack of evidence. In fact, despite a more gradual and longer deployment than usually done with metallic stents is both suggested by the producer and endorsed in a recent expert review [13], no direct experimental evidence was available, yet.

## Conclusions

A correct and careful implantation technique including a 30”-long balloon dilation is key for a successful implantation, and to achieve maximal scaffold expansion. Proper stent deployment minimizes the prevalence of post-PCI residual stenoses. BVSs are a major breakthrough in the field of coronary interventions.

### Limitations

The main limitation of the present study is the lack of intracoronary imaging. For this reason, we couldn’t evaluate malapposition. However, such evaluation would have required removal of the balloon catheter after scaffold deployment, introduction of the imaging catheter, and another exchange to the balloon catheter to perform the 30”-long dilation. Finally, after removal of the catheter a new run with the imaging catheter would make required the introduction of a further intracoronary device. In other words, this would have required a significant prolongation of both total procedural time and radiation dose. However, the specific aim of the present study was to evaluate the internal coronary diameter at the different balloon inflation steps, therefore the QCA is appropriate technology to this end. Finally, since optimal lesion pre-dilatation was an inclusion criterium for the present study our conclusions cannot be generalized to a different population. In fact, despite BVS implantation is not recommended in lesions with suboptimal response to pre-dilatation, several operators are implanting BVSs in unselected lesions.

## Additional files

Additional file 1: Figure S1. Inter-observer variability of QCA measurement. QCA = quantitative coronary angiography. (TIFF 2932 kb) Additional file 2: Figure S2. Delta change of MR-Ratio after the 30”-long balloon dilation in study subgroups. stable ACS = acute coronary syndrome; CAD = coronary artery disease; Balloon Ratio = pre-dilatation balloon diameter-to-nominal scaffold diameter Ratio; RVD = reference vessel diameter. (TIFF 2932 kb)

## Abbreviations

ACS: Acute coronary syndrome
ATM: Atmosphere
Balloon Ratio: pre-dilatation balloon diameter-to-nominal scaffold diameter Ratio
BVS: Bioresorbable vascular scaffolds
CAD: Coronary artery disease
MLD: Minimal luminal diameter
MSD: Minimal scaffold diameter
MR-Ratio: MLD-to-nominal scaffold diameter Ratio
NSD: Nominal scaffold diameter
NSTEMI: Non-ST-elevation Myocardial Infarction
PLLA: poly-L-lactic acid
PDLLA: poly-D,L-lactide
PSI: Pound-force per square inch
QCA: Quantitative coronary angiography
RSD: Reference scaffold diameter
RVD: Reference vessel diameter
STEMI: ST-elevation Myocardial Infarction
TLF: Target lesion failure
TVF: Target vessel failure
UA: Unstable angina
