# Supplementary figures and images for: The duration of balloon inflation affects the luminal diameter of coronary segments after bioresorbable vascular scaffolds deployment
Source: BMC Cardiovasc Disord. 2015 Dec 11;15:169. doi: 10.1186/s12872-015-0163-5 (PMC4676860; doi:10.1186/s12872-015-0163-5)

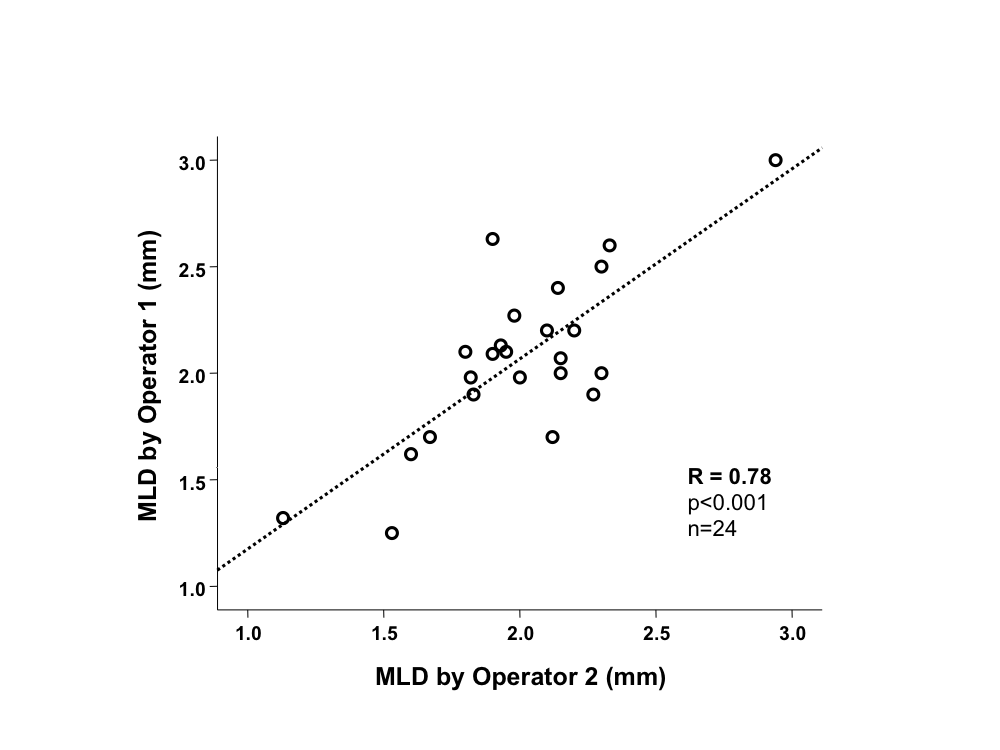

Supplement: Additional file 1: Figure S1. — Inter-observer variability of QCA measurement. QCA = quantitative coronary angiography. (TIFF 2932 kb) [file 12872_2015_163_MOESM1_ESM.tiff]

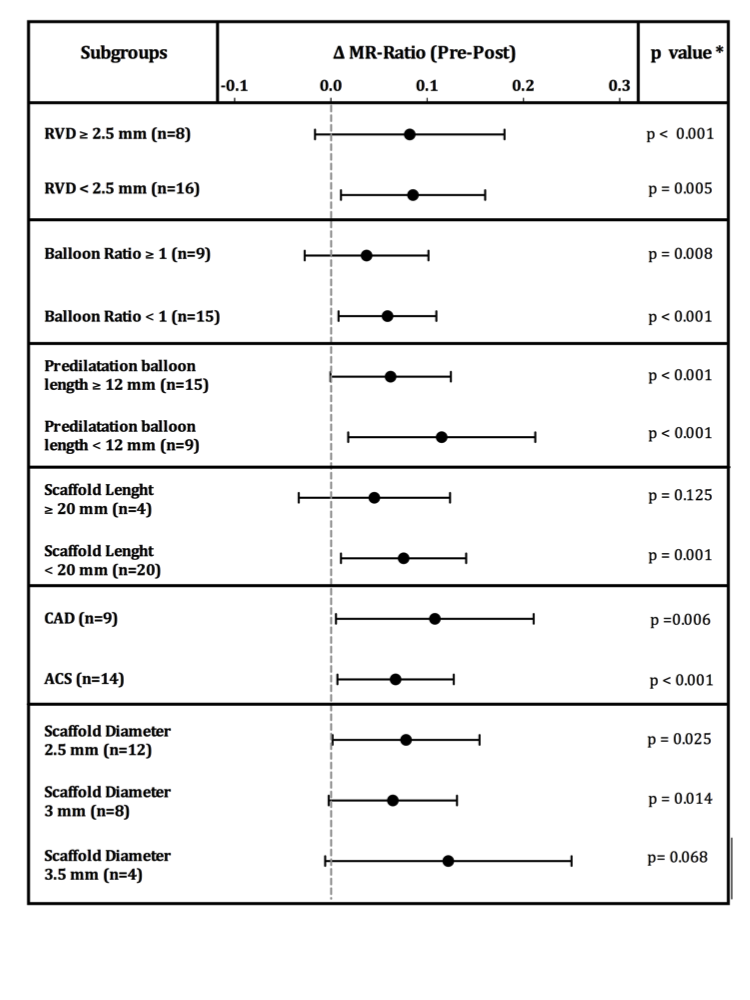

Supplement: Additional file 2: Figure S2. — Delta change of MR-Ratio after the 30”-long balloon dilation in study subgroups. stable ACS = acute coronary syndrome; CAD = coronary artery disease; Balloon Ratio = pre-dilatation balloon diameter-to-nominal scaffold diameter Ratio; RVD = reference vessel diameter. (TIFF 2932 kb) [file 12872_2015_163_MOESM2_ESM.tiff]
